# Unveiling “The Bellomo Effect”: A tribute from Professor Rinaldo Bellomo’s Research Fellow Family

**DOI:** 10.1016/j.ccrj.2025.100141

**Published:** 2025-10-14

**Authors:** International Research Fellows Group, Anis Chaba, Alessandro Caroli, Jonathan Nübel, Yukiko Hikasa, Nuanprae Kitisin, Akinori Maeda, Sofia Spano, Ary Serpa Neto

**Affiliations:** a Department of Intensive Care, Austin Hospital, Heidelberg, VIC, Australia; b Australian and New Zealand Intensive Care Research Centre (ANZIC-RC), School of Public Health and Preventive Medicine, Monash University, Melbourne, Australia; c Intensive Care Unit, Tenon Hospital, Assistance Publique des Hôpitaux de Paris (APHP), Paris, France; d Dipartimento di Scienze dell’Emergenza, Anestesiologiche e della Rianimazione, Fondazione Policlinico Universitario A. Gemelli IRCCS, Cattolica del Sacro Cuore Largo A. Gemelli 8, Rome 00168, Italy; e Department of Cardiology, University Hospital Heart Centre Brandenburg, Brandenburg Medical School (MHB) Theodor Fontane, Bernau, Germany; f Department of Anesthesiology, Faculty of Medicine, Siriraj Hospital, Mahidol University, Bangkok, Thailand; g Department of Critical Care, Melbourne Medical School, University of Melbourne, Austin Hospital, Melbourne, Australia; h Data Analytics Research and Evaluation (DARE) Centre, Austin Hospital, Melbourne, Australia; i Department of Intensive Care, Royal Melbourne Hospital, Parkville, VIC, Australia

Dear Editor,

We would like to comment on an extraordinary phenomenon which we term “The Bellomo Effect” that had been shaping the world of intensive care for the past 40 years. Much like the “butterfly effect” which is the idea that one event can have far-reaching ripple effects over time and in other places, the “Bellomo Effect” similarly refers to the personal and professional effects experienced by those mentored by Professor Rinaldo Bellomo.

It all started in a very small office in Melbourne, Australia, a place where a research team resembles a family and people would feel at home when coming from all over the world to learn from Professor Rinaldo Bellomo, a clinician-researcher they had heard so much about. They came to learn how to conduct rigorous clinical trials, or so they thought. In reality, they ended up learning so much more.

Research fellows often testified of how caring Rinaldo, as he preferred to be called, was. But as he would often cite “It is a mistake to theorise before one has data. Insensibly one begins to twist facts to suit theories, instead of theories to suit facts”.^1^ Accordingly, we conducted an international survey to explore the true extent of his impact. We aimed to test the primary hypothesis that Rinaldo spread his rigour and scientific ethic over the majority of fellows he met. Moreover, we sought to test the secondary hypothesis that he was a profoundly caring mentor who shaped research careers as well as entire professional journeys. Finally, we tested an exploratory hypothesis that beyond shaping medical literature, he built a research family whose bonds have remained unbreakable across time and continents.

## 1 Methods

### 1.1 Oversight and study setting

We conducted an online survey in accordance with all relevant ethical standards and current recommendations.^2^ In conducting the survey, all individuals contacted by email were informed that their responses might be included in a scientific publication, and participation in the survey was considered as informed consent. As the survey involved only colleagues and collaborators, ethics committee approval was not deemed necessary.

### 1.2 Study population and data collection

We purposefully sought international research fellows who had been mentored by Rinaldo at the Department of Intensive Care of Austin Hospital between 1998 and 2025. Research fellows were identified through institutional records, Rinaldo’s current curriculum vitae, and publicly available academic profiles.

All identified research fellows were invited to participate in the survey, which was open for responses for 4 weeks. The survey was pilot tested among five most recent fellows mentored by Rinaldo and then distributed via email invitation, using Microsoft Forms (shown in the Supplemental material). The questionnaire comprised open-ended questions covering the following domains: the influence of Rinaldo on participants’ emotional well-being, mentorship experience, and long-term professional development. All responses were collected anonymously, except for self-identified quotes by the respondent.

Additionally, to illustrate the academic impact of the “Bellomo effect”, publication record from all research fellows from 1998 to 2025 as indexed in PubMed, were collected.

### 1.3 Statistical analysis

Descriptive statistics were used to summarise geographic data and publication counts. Changes in publication output across time periods were analysed using a linear mixed model treating fellows as a random effect. To capture the collective perception of Rinaldo’s character and influence, the survey responses from mentees describing Rinaldo’s mentorship were harmonised into a word map. Quotations either spoken by Rinaldo or favoured by him were cited to provide insights into his character and legacies. All statistical analyses were performed using R version 4.4.1 (R Core Team. 2024. R: A language and environment for statistical computing. R Foundation for Statistical Computing, Vienna, Austria).

## 2 Results

### 2.1 Scientific rigour and ethical research

**Fig. 1:**
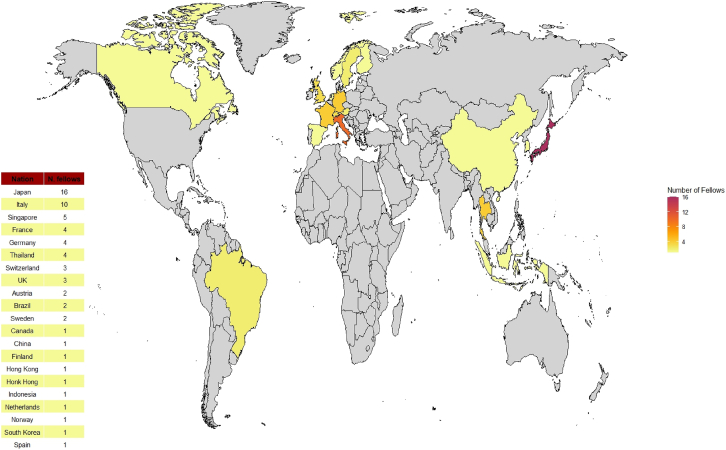
Global distribution of international research fellows mentored by Professor Rinaldo Bellomo. World map showing the countries of origin of the international Fellows who were mentored by Professor Rinaldo Bellomo. A total of 66 research fellows from 21 countries attended a period of clinical research at the Department of Intensive Care, Austin Hospital, in Melbourne, Australia, between 1998 and 2024. Japan, Italy, and Singapore were the top three contributing countries. The colour gradient indicates the number of fellows per country, with darker shades representing a higher number of mentees.

From 1998 to 2025, Rinaldo mentored 66 fellows from 21 countries (Fig. 1). Together, they covered a multitude of medical fields which included critical care nephrology, sepsis and inflammatory pathophysiology, and clinical trials in intensive care medicine. Rinaldo always applied the same approach to answering research questions: set the environment with a systematic review, conduct an observational study, design a pilot trial, and, if all lights are green, run a large randomised controlled trial. Rinaldo was a “dogma hunter”: “question everything, ask the question then question the answer”. He was equally intransigent on the quality analyses as he was on the narrative. “We cannot send a paper to the *New England Journal of Medicine* wearing sandals and socks”. He always found the proper outfit for each of our studies, always with the aim of “bringing the reader with us”. Every scientific question was methodologically stated on the backbone of “Does this actually help the patient?” Rinaldo was fully aware of statistical modelling limitations and regularly cited Box’s famous quote: “All models are wrong, but some are useful”. He religiously asked for the data to “be tortured until they confess” and wanted each result to be challenged as much as possible. Rinaldo made sure every study conducted found a place in the best journal possible, leaving no unpublished results.

### 2.2 Mentorship and impact on academic careers

Rinaldo’s 15-m^2^ office without a single window was an incandescent source of energy. He was always sitting in front of his computer on a tiny unergonomic office chair covered with a 10 × 20-cm white old pillow. On his left, there was a wall where pictures of his family were in a peaceful harmony with research plans. Behind him, there were wall shelves full of medical books that he wrote. Below, stood a table with multiple organised stacks of papers. Each one marked a fellow, displaying projects they were working on and often what was to come over the next 10 years.

**Fig. 2:**
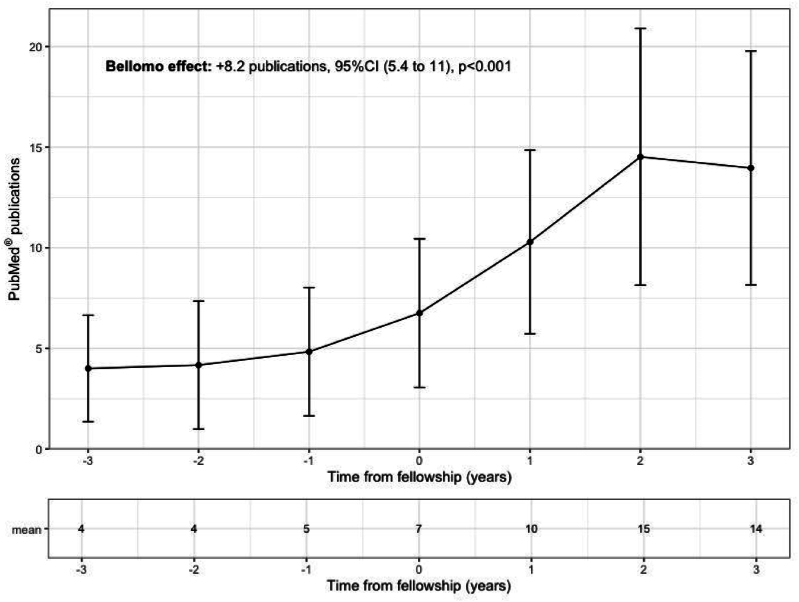
Mean PubMed© publication according to the time from fellowship with Rinaldo started. All the publications of 66 fellows mentored by Rinaldo at the Austin Hospital, Melbourne, Australia, were included. A linear mixed model was used to model the mean publication per fellow overtime, accounting for a random intercept per fellow. CI: confidence interval.

Rinaldo impacted many scientific careers. As displayed in Fig. 2, starting a research fellowship at Austin Hospital was associated with an +8.2 publications increase (95% confidence interval: [5.4 to 11.0], p < 0.001). More than publications-only, Rinaldo encouraged fellows to become Professors, clinician-researchers, and leaders in their home countries. Also, Rinaldo always wanted to make sure fellows found the right balance between their academic ambitions and a cheerful private life.

He would keep enquiring, in his iconic tone, “Are you happy?”, “Happy wife, happy life”, “Please go home, it is too late”, and “You should visit (random place in Australia)”. One fellow remembered this quote from him: “If you prioritise work too much over your personal life, you risk not only damaging your private life but also undermining the research you’ve placed above it”.

Now, as professors or leaders, fellows report that they continue to apply his lessons: how to mentor, how to communicate, and how to approach uncertainty.

### 2.3 Research family

The research team grew naturally under Rinaldo’s leadership and became one of his most enduring legacies. At its heart were the “Fantastic Four” (Rinaldo, Glenn, Leah, and Helen), a stable core known for their warmth, humour, and commitment to helping fellows grow. Each year, the team expanded with new fellows from around the world, forming a global family that kept growing without losing its bonds.

**Fig. 3:**
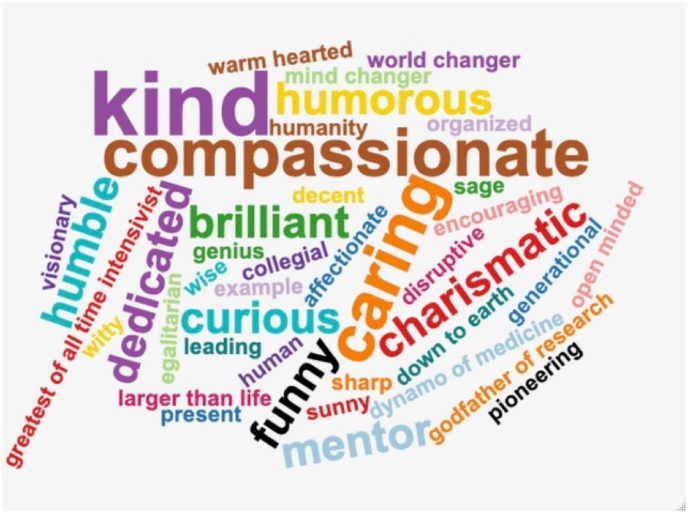
Word map of the adjectives used by the research Fellows to describe Professor Rinaldo Bellomo’s character and spirit.

Rinaldo often randomly popped up in the research office for a coffee/chocolate break with a joke, a history anecdote, or a political fun fact, often all mixed in one but always astutely linked to physiology or research projects. Under his watch, noradrenaline, vasopressin, and angiotensin II became ATHOS, PORTOS, and ARAMIS, respectively, while paracetamol overdose was summarised by the phrase by Paracelsus’ “There are no poisons, only poison doses”. During these unique moments, Rinaldo was charismatic, unpredictable, curious, unconventional, and somewhat normal. The main words used by fellows to describe Rinaldo are displayed in Fig. 3.

One of the most extraordinary things was his ability to make every unsure, self-doubting fellow feel confident in who they were and what they were meant to achieve. “You made it through immigration and the Australian Health Practitioner Regulation Agency. You’re here, and that alone proves you’re extraordinary”, he would say.

It was thanks to this gift, and everything else we have shared, that Rinaldo managed to leave a little piece of himself in each fellow, now scattered across the world.

## 3 Discussion

### 3.1 Key findings

Our study has four key findings. First, we demonstrated that Rinaldo was seen as a kind, caring, and compassionate mentor by the vast majority of his research fellows. Second, we found that Rinaldo was seen as an example of scientific rigour with a matchless ethic of research. Third, we elicited Rinaldo’s profound influence on his fellows and his capability of elevating their professional trajectories while sustaining their growth as clinicians, as researchers, and as human beings. Finally, we showed that Rinaldo built not only a research empire but an inseparable family all over the world.

### 3.2 Relationship with previous studies

Rinaldo’s loss prompted a powerful global outpouring of respect, admiration, and love, leading to the publication of numerous articles across international journals.[3], [4], [5], [6], [7], [8] All of them are consistent with our main findings.

Forni et al. defined Rinaldo as a “restless architect of modern intensive care”. In the article, they emphasise how Rinaldo’s visionary ideas led to the foundation of critical care nephrology and how his transformative contributions enabled the first consensus definition of acute kidney injury.^3^ Kellum et al. reminded us that he was the first intensive care physician to be acknowledged by Clarivate Analytics as one of the most influential scientific minds of our time, one among many honours he received, and that his body of work encompasses countless impactful publications.^4^ These achievements would have been unattainable without his commitment to methodological rigour, the pursuit of high-quality data, and the search for patient-centred scientific questions.

Landoni and Baiardo Redaelli highlight Rinaldo’s scientific heritage by describing five milestone concepts that converge with the strategy for scientific research previously described.^5^ Neto and Young reported how “his office door was always open, a literal and symbolic invitation for anyone, regardless of seniority, to step in, discuss, learn, and collaborate”.^7^ These concepts align with our findings and show how profoundly Rinaldo exemplified the roles of “mentor” and “educator” in their true etymological sense.

Landoni and Baiardo Redaelli further document his expansive global network across more than 70 countries.^5^ Moreover, Neto and Figueira Salluh, together with Marshall, describe Rinaldo’s strong commitment to fostering collaboration among researchers, promoting journal cooperation to amplify scientific impact, and his remarkable ability to unite people.^6^^,^^8^ These are undeniable proofs of his ability to break barriers and build long-lasting bridges.

Finally, in every editorial, his role as the builder of people and ideas and his kindness, integrity, humbleness, honesty, extensive general culture, and genuine care for his colleagues, fellows, and family are firmly affirmed.

### 3.3 Implications of study findings

Our findings have several implications. First, they imply that Rinaldo’s mentorship had a lasting impact on both the personal and professional lives of his fellows. Beyond scientific achievement, his example fostered a culture of compassion, collaboration, and integrity. The findings underscore the importance of strong mentorship in shaping not just careers but also the values and community spirit within academic medicine. This enduring influence not only leads to deep grief over the incredible loss but also leaves behind a strong, lasting network that we refer to here as *“The Fellow Family”*.

### 3.4 Strengths and limitations

Our study has several strengths. To the best of our knowledge, this is the first prospective study investigating Rinaldo’s impact on people mentored by him across the globe. As someone once said, each of us carries a unique image of Rinaldo, shaped by our personal relationship with him. Through this editorial, we sought to bring those images together, creating a collective portrait drawn from the memories of many fellows mentored by him over more than three decades.

However, we acknowledge some limitations. First, the survey included only international fellows trained at Austin Hospital, although Rinaldo’s influence extended far beyond this group. However, our sample alone spans a wide range of countries, specialties, and generations, offering valuable insights into his global influence. Second, not all former fellows could be reached, as up-to-date contact details were unavailable for some. However, we were still able to email the vast majority of them. Finally, given the recency of his passing, some individuals may have found it too personal or emotionally difficult to respond. However, their silence has been embraced by the rest of the research family, and we hope, in some way, to have given voice also to their feelings through this tribute.

## 4 Conclusion

Our findings provide evidence for the profound influence that Rinaldo had on generations of fellows, both scientifically and personally. This unique combination of academic rigour and human connection has often been described as “The Bellomo Effect”.

Such an impact is barely captured by data alone. Yet, perhaps the greatest recognition any mentor can receive is to comprehend the extent to which they have shaped the lives and values of others. All we can hope is that you, Rinaldo, already knew all of this before you left. And knowing you, you probably did, just as you always seemed to know everything ahead of time. But just to be sure, we wanted to leave in black and white a trace of everything you have been to us and a testimony for those who never had the luck to know you in person.

You lived in an exceptionally warped time dimension, where you somehow made room for everyone you cared about and every single one of your passions. We honestly wonder if there was anything in this world you were not curious about. None of your endless energy was ever spent on judging, getting angry, or trying to stand above anyone. There was no need—you were galaxies ahead, and yet the most present in daily life. You knew more than anyone we have ever met, but it never made you arrogant. You had strong, inspiring opinions on everything, yet they never kept you from listening. You were the busiest person we have ever known, and somehow the most available.

You were there, every day, for everyone. For every knock at your door, there was always a “come on in!” What followed was usually 5 min of discussing solutions to whatever urgent issue we had (Oh, you were good at it) and an hour reflecting on something that had happened, who knows when, in the history of humanity. No one ever really understood how the conversation got there but left so enriched they did not even wonder. Time, after leaving your door, was usually spent putting into action the ideas sparked in those first 5 min of conversation—Remember? The reason we had knocked in the first place.

All you ever wanted was for people to find their path, achieve what they dreamed of, feel safe, and have fun. You were there to laugh, never to weigh things down. You were there to guide, not to choose on anyone’s behalf. No one once heard you telling someone what their priorities should be, except to try to be happy.

In a world where people often expect more than they give, you—humble giant—walked the other way. As always. And so will we.

You changed the eyes of everyone who had been lucky enough to walk beside you, even briefly. You kept so many promises. We will keep them for you now.

Thank you for our new life,

The Fellow Family.

## Funding

This research did not receive any specific grant from funding agencies in the public, commercial, or not-for-profit sectors.

## CRediT authorship contribution statement

Conceptualisation: All authors. Methodology: All authors. Formal analysis: Anis Chaba, Jonathan Nubel, Alessandro Caroli. Validation: Anis Chaba, Sofia Spano. Supervision: Ary Serpa Neto. Writing-original draft: Sofia Spano. Writing-review and editing: All authors.

## Conflict of interest

We wish to declare that, as international research fellows, we all have a close personal relationship with Professor Rinaldo Bellomo, which naturally reflects the nature and perspective of this invited editorial about him. Apart from this, we have no financial or other conflicts of interest to declare.
