# Supplementary material for: Unveiling “The Bellomo Effect”: A tribute from Professor Rinaldo Bellomo’s Research Fellow Family
Source: Crit Care Resusc. 2025 Oct 14;27(3):100141. doi: 10.1016/j.ccrj.2025.100141 (PMC12847625; doi:10.1016/j.ccrj.2025.100141)

**Unveiling “The Bellomo Effect”**

**A Tribute from Professor Rinaldo Bellomo’s Fellow Family**

**Supplementary**

[Survey Design and Implementation 1](#_Toc204001014)

[Questions of the Online Survey: 1](#_Toc204001015)

[Summarized answers with selected anonymized responses 2](#_Toc204001016)

# **Survey Design and Implementation**

The online survey was available from 20 May 2025 to 17 June 2025. A total of 56 Fellows were contacted via email; 29 completed responses were received. The average time required to complete the survey was 34 minutes. The original survey questions are listed below.

Participants were instructed to select three out of the eight questions, with question 1 being mandatory, to contribute to the generation of **Figure 3**.

# **Questions of the Online Survey:**

1. If you had to describe Rinaldo in three words, what would they be?
2. Besides his remarkable achievements as a researcher and clinician, what personal side of him left a strong impression on you?
3. What was Rinaldo’s greatest lesson to you - scientific, personal, or both?
4. Is there a specific situation or conversation with Rinaldo that stayed with you? Please describe it briefly.
5. In what ways does Rinaldo continue to influence your professional or personal life since your fellowship?
6. What does being part of the 'Fellow Family' at Austin mean to you—what stays with you from that time?
7. If you could say something to Rinaldo now, what would you like to tell him?
8. Is there anything else you would like to share?

# **Summarized answers with selected anonymized responses**

**1. Besides his remarkable achievements as a researcher and clinician, what personal side of him left a strong impression on you?**

**Summary:**
Respondents consistently described Rinaldo as deeply human, kind, supportive and intellectually curious. He was remembered not only for his brilliance and mentorship, but for his genuine interest in the lives and happiness of his fellows. His sense of humour, warmth and generosity with his time left a profound impact.

**Selected anonymized responses:**

- “He was a family man who always showed a sincere interest in my personal life. He knew it was more important to have a happy family than to give every living hour to your job.”
- “Rinaldo combined exceptional intellect with a rare ability to make others feel valued. He celebrated our successes as if they were his own and was always a trustworthy mentor and a big brother we could rely on.”
- “His generosity with his time, his sense of humour, and his humility made him an inspiring leader. He always found time to talk about life, not just science.”

**2. What was Rinaldo’s greatest lesson to you - scientific, personal, or both?**

**Summary:**
Rinaldo’s greatest lessons centred on scientific honesty, mentorship, curiosity and balancing professional achievement with personal well-being. Fellow’s recall being inspired to be both better scientists and better people.

**Selected anonymized responses:**

- “Never cheat! Must publish papers. Happy wife, happy life.”
- “You cannot be a good doctor if you are not a good person first.”
- “He made us realize the importance of mentorship. It may be draining, but it is very fulfilling. I carry that belief in my own work with others.”

**3. Is there a specific situation or conversation with Rinaldo that stayed with you? Please describe it briefly.**

**Summary:**
Fellows shared stories of pivotal moments: support during challenging times, lessons in integrity, and personal encouragement. Many recall small but powerful gestures that showed Rinaldo’s care and humanity.

**Selected anonymized responses:**

- “I was struggling with self-doubt, and he reminded me that just making it to the Austin was an achievement. He made me feel like I belonged.”
- “Once, after joking about ‘making up a p-value,’ he became serious and told me, ‘ as clinical researchers, our reputation is all we have, you can never cheat.’ I use this lesson with my own fellows now.”
- “When I broke down in his office about missing my family, he said he had given me work to distract me, showing how much he cared about our well-being.”

**4. In what ways does Rinaldo continue to influence your professional or personal life since your fellowship?**

**Summary:**
Rinaldo’s influence persists in fellows’ professional practices, teaching habits, and approach to research and mentorship. Many now model their own behaviour on his commitment to excellence, integrity, and human connection.

**Selected anonymized responses:**

- “Rinaldo answered almost every e-mail within 24 hours. I now do the same and teach my students this habit.”
- “There isn't a week where I don't use one of Rinaldo's quotes either with my own fellows or during rounds.”
- “His blend of intellectual curiosity, wisdom, humour, and generosity set a model I still strive to follow. I often find myself thinking, What would Rinaldo do?”

**5. What does being part of the 'Fellow Family' at Austin mean to you - what stays with you from that time?**

**Summary:**
The “Fellow Family” is described as a unique, supportive, and diverse community bound by Rinaldo’s leadership and example. This experience fostered lifelong friendships, professional growth, and a deep sense of belonging.

**Selected anonymized responses:**

- “Being part of the ‘Fellow Family’ at Austin meant growing together - supporting each other’s ideas, papers, and progress. We were united by a deep connection to Rinaldo and a shared commitment to something bigger than ourselves.”
- “The fellow family is part of Rinaldo’s magic. It keeps expanding around the world without breaking bonds. The Research Office has been home for me on the opposite side of the world.”
- “Being there gave me incredible strength and self-confidence I never had before. My life changed after Austin in almost every aspect.”

**6. If you could say something to Rinaldo now, what would you like to tell him?**

**Summary:**
Most would express profound gratitude, a sense of loss, and a commitment to carry forward his legacy in their own work and lives. Many expressed how deeply Rinaldo changed them.

Selected anonymized responses:

- “Thank you for seeing potential in all of us, for challenging us, and for leading with both brilliance and heart. Your influence runs deep, and your spirit continues to guide so much of what we do.”
- “We will continue your legacy. Please know that you have made me a better person. I had always wanted to return to Austin to tell you how much you influenced me.”
- “Thank you for giving me one of the best opportunities in my life. You became my role model - not just in how to conduct research, but in how to lead with passion and integrity.”

**7. Is there anything else you would like to share?**

**Summary:**
Additional comments highlighted the importance of family, the supportive Austin community, gratitude toward Rinaldo’s colleagues, and the profound privilege of working alongside him. Many reflected on how even small interactions with Rinaldo left a lasting mark.

**Selected anonymized responses:**

- “We took photos with the whole family specifically to send to Rinaldo, because he loved those updates from around the world. I did not get a chance to send them, which just reminds me that you should send important emails within 24 hours.”
- “He encouraged us to bring our families to conferences and share quality time, even as we worked. He was a fantastic human being. We will always celebrate his life.”
- “Rinaldo wouldn't have been Rinaldo without all the tremendous people who shared his daily professional life. The office team welcomed fellows with open arms, leading us all to the best achievements and moments.”

# **Collage of the Research Fellow Family**


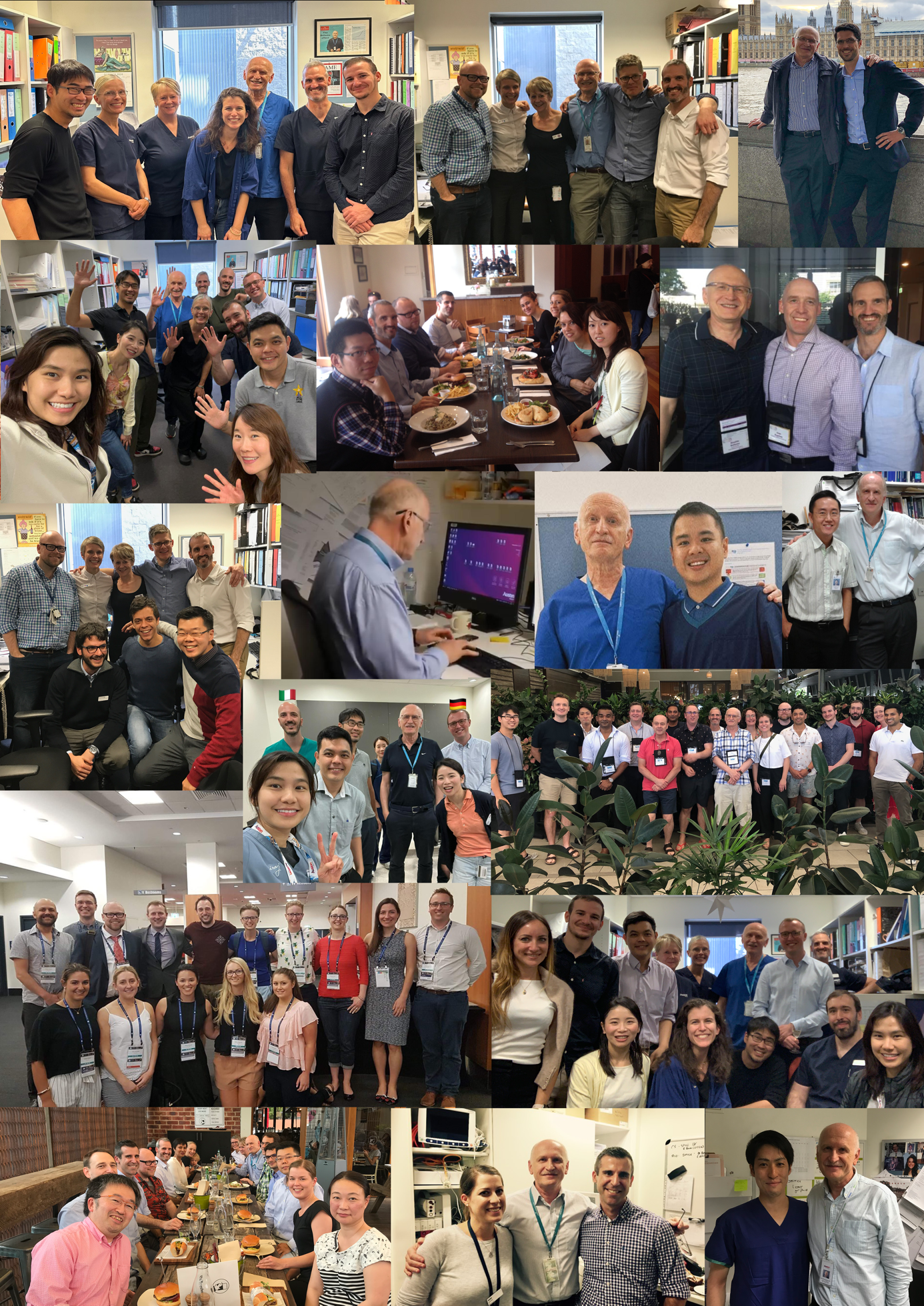

Supplement: Multimedia component 1 [file mmc1.docx]
